# Structure of the Altitude Adapted Hemoglobin of Guinea Pig in the R2-State

**DOI:** 10.1371/journal.pone.0012389

**Published:** 2010-08-24

**Authors:** Bruno Pairet, Elmar Jaenicke

**Affiliations:** Institut für Molekulare Biophysik, Johannes Gutenberg Universität, Mainz, Germany; Massachusetts Institute of Technology, United States of America

## Abstract

**Background:**

Guinea pigs are considered to be genetically adapted to a high altitude environment based on the consistent finding of a high oxygen affinity of their blood.

**Methodology/Principal Findings:**

The crystal structure of guinea pig hemoglobin at 1.8 Å resolution suggests that the increased oxygen affinity of guinea pig hemoglobin can be explained by two factors, namely a decreased stability of the T-state and an increased stability of the R2-state. The destabilization of the T-state can be related to the substitution of a highly conserved proline (P44) to histidine (H44) in the α-subunit, which causes a steric hindrance with H97 of the β-subunit in the switch region. The stabilization of the R2-state is caused by two additional salt bridges at the β1/β2 interface.

**Conclusions/Significance:**

Both factors together are supposed to serve to shift the equilibrium between the conformational states towards the high affinity relaxed states resulting in an increased oxygen affinity.

## Introduction

Life at high altitudes is markedly characterized by low oxygen availability, which challenges aerobic metabolism. Vertebrates show a remarkable ability to adapt to life under these conditions by developing several strategies both on the organismic and molecular level to alleviate the effects of low oxygen availability as recently reviewed [1], [2], [3]. Hemoglobin, the oxygen transport molecule in the blood of vertebrates, is the most important target for adaptations on the molecular level.

Hemoglobins in vertebrates show the typical globin fold accommodating a heme group which reversibly binds oxygen to its central iron atom [4], [5], [6]. In vertebrate erythrocytes hemoglobins are usually present as tetramers (α_2_β_2_) of two α-chains and two β-chains, each chain with a molecular mass of ∼15 kDa. An important step in hemoglobin evolution was oligomerization through which cooperative oxygen binding and allosteric control of binding properties were acquired. Both, cooperativity and allosteric control in conjunction, render hemoglobin a highly versatile oxygen carrier whose binding properties can be tuned over a wide range by allosteric effectors such as protons and 2,3 bisphosphoglycerate [5], [6].

Cooperative oxygen binding has been successfully explained by the two-state model developed by Monod, Wyman and Changeux based on the structures of the T-state (tense, unligated) and R-state (relaxed, ligated) observed in hemoglobin crystals [7], [8], [9]. Decades later a second quaternary structure was described for liganded hemoglobin. This quaternary structure, named R2-state, showed spatial differences relative to the previously known R-state that equaled in magnitude the spatial differences between R- and T-state [10]. Initially the R2-state was thought to be a stable intermediate along a T↔R2↔R quaternary transition pathway [10], later the R2-state was suggested to be the endpoint along the quaternary structure transition T↔R↔R2 [11], [12]. Determination of the quaternary structure of normal adult hemoglobin with the ligand carbonmonoxide under almost physiological conditions by NMR, revealed that the “in solution” structure of hemoglobin is a dynamic intermediate between the two quaternary states R and R2 [13].

Several strategies for adaptation to high altitudes on the genetic level are known with repect to hemoglobin structure. The hemoglobin of new world camelids such as llama, guanaco and vicuña, which live in altitudes up to 5000 m, has a partly degenerated binding site for the allosteric effector 2,3-bisphosphoglycerate caused by a His2→Asn mutation on the β-subunit, which removes two of the seven contacts between the hemoglobin molecule and its allosteric effector 2,3-bisphosphoglycerate thereby increasing oxygen affinity [14], [15], [16]. The hemoglobin of barheaded geese, which fly at altitudes up to 9000 m, features an amino acid exchange at the α_1_β_1_ interface increasing oxygen affinity by a release of tension in the hemoglobin molecule [17]. Recently in deer mice several hemoglobin isoforms and their differential expression depending on oxygen availability were characterized. The oxygen binding characteristics of the isoforms in this case seems to depend on several concomitant amino acid exchanges within the hemoglobin molecule [18].

Guinea pigs are an important live-stock in the Andean region and considered to be genetically adapted to a high altitude environment. This is based on the consistent finding of a high oxygen affinity of their blood and the observation that guinea pigs develop only moderate erythrocytosis when exposed to chronic hypoxia. Both characteristics are not restricted to guinea pig living at high altitude, but are also found in guinea pigs which were born and raised at sea level [19]. Typical guinea pig blood has a p_50_ of 25 Torr at pH 7.4 [20], [21], [22]. The sensitivity of guinea pig towards the allosteric effector 2,3-bisphosphoglycerate is normal [23]. The oxygen affininity of guinea pig hemoglobin is high in comparison with other animals of equivalent size and lifestyle such as rat, shrew, hedgehog and deer mice, which have p_50_ values of around 36 Torr at pH 7.4 [21], [24], [25].

Guinea pig blood contains one major hemoglobin component and two minor hemoglobin components, which are only present in very small quantities [26]. The sequence of the major hemoglobin component of guinea pig is known [26]. However, lacking a high resolution crystal structure of guinea pig hemoglobin, the molecular basis for the high oxygen affinity of guinea pig hemoglobin remains unknown.

We have recently reported the crystallization of the major component of guinea pig hemoglobin in the met-state and now present the first structure of guinea pig hemoglobin at 1.8 Å resolution [27].

## Methods

### Isolation and purification

Guinea pig (*Cavia porcellus*) blood in Alsever solution was obtained from Charles River Laboratories (Sulzfeld, Germany). Guinea pig hemoglobin was isolated and purified according to Paoli and Nagai (2004) based on the original protocol of Perutz [5], [28]. Briefly, erythrocytes were separated from plasma by centrifugation at 100 g for 15 min. Then erythrocytes were washed with isotonic saline (0.9% NaCl) and subsequently lysed by adding an equal amount of water, resulting in a release of hemoglobin from the cells. After addition of NaCl to a final concentration of 3%, cell debris was removed by centrifugation. As hemoglobin represents 98% of protein in the hemolysate, no further purification was necessary.

### Crystallization

Crystallization of guinea pig hemoglobin was performed by hanging-drop vapor diffusion at 20°C. The drops contained 5 µl hemoglobin solution with a concentration of 10 mg/ml and were mixed with 5 µl reservoir solution. Then the drops were equilibrated against 1.0 ml reservoir solution (2.6 M (NH_4_)_2_SO_4_, 100 mM sodium phosphate buffer, pH 6.5).

### Data collection and processing

Prior to data collection the crystals were soaked in mother liquor containing 25% glycerol as cryoprotectant. Crystals were then flashed cooled in the gas stream of a cryostream system (Oxford Cryosystems, Oxford, United Kingdom), with a nitrogen gas temperature of 100 K. Data was collected using a Microstar rotating anode (Bruker AXS, Karlsruhe, Germany) and a “mar345” image plate detector (MARresearch, Norderstedt, Germany). Data was collected for 360 ° with an increment of 1.5 ° and a crystal to detector distance of 120 mm. Data was collected up to a resolution of 1.8 Å and processed with the XDS program package (Version: December 6th 2007) [29]. The space group was determined using the program POINTLESS from the CCP4 program suite [30].

### Structure solution and refinement

Crystal parameters have been reported before [27]. The structure solution was obtained by molecular replacement with carbonmonoxy horse hemoglobin (PDB-code: 2D5X) as starting model, using the program PHASER implemented in the CCP4 suite [31]. The structure was refined using the program Coot/REFMAC [32]. Data collection and refinement statistics for the final model are presented in Table 1. The final model and structure factor were deposited in the Protein Data Bank with accession code 3HYU.

**Table 1:** Crystallographic parameters.

**Diffraction data**	
Wavelength, Å	1.54
Space group	C 222_1_
a, Å	84.54
b, Å	99.95
c, Å	82.72
Resolution range, Å	19.6-1.67 (1.71-1.67)
No. of measurements	472687 (55630)
No. of unique reflections	36378 (5585)
Completeness, %	99.3 (92.1)
R_MERGE_	0.052 (0.195)
<I>/<σ>†	35.8 (9.95)
**Refinement**	
R_cryst_	0.179
R_free_ *	0.203
No. of protein atoms	2308
No. of water molecules	229
**rms deviation from ideality**	
Bonds, Å	0.011
Angles, °	1.165
**Average ** ***B*** ** value, Å**	
All atoms	12.37
Main chain	10.03
Side chain and water	14.31
**Ramachandran plot**	
Residues in most favorable regions, %	98.6
Residues in additional allowed regions, %	1.4

**Data collection and refinement statistics**.

Numbers in parentheses refer to the highest resolution shell.

*Test set size was 5% of reflections.

† *<I>/<σ>*  =  ratio between the mean intensity and the mean error of the intensity.

Molecular graphics were produced using PyMOL Molecular Graphics System (DeLano Scientific, USA).

## Results and Discussion

### Overall description of the structure

**Figure 1:**
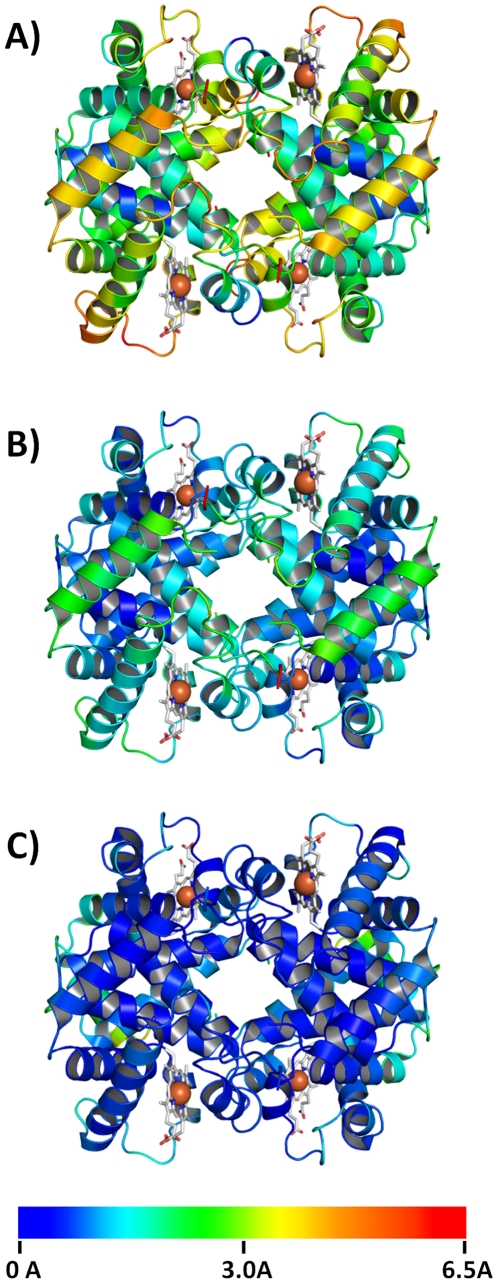
Conformational state of guinea pig hemoglobin. The structure of guinea pig hemoglobin (PDB-code: 3HYU) was superimposed with the structures of three conformational states of human hemoglobin by their C_α_-atoms. The guinea pig hemoglobin structure is shown in cartoon representation, which is colored according to the distance between corresponding C_α_-atoms in guinea pig hemoglobin and the respective conformational state of human hemoglobin in (A) T-state (PDB-code: 1A3N, [36]), (B) R-state (PDB-code: 1HHO, [37]) and (C) R2-state (PDB-code: 1BBB, [10]). Distances between C_α_-atoms clearly show that guinea pig hemoglobin crystallizes in the R2-state (C). Color coding of C_α_-atoms distances was made according to the colors given in the bar below.

The structure of guinea pig hemoglobin is a typical vertebrate hemoglobin tetramer made up from two α-subunits and two β-subunits of 141 and 146 amino acids, respectively (Fig. 1). The electron density clearly confirmed the sequence, which had previously been reported for the major hemoglobin component [26]. The α-chain of guinea pig hemoglobin shares 75% identical amino acids with human hemoglobin, while the β-chain is identical with its counterpart in human hemoglobin in 65% of the positions. Absorption spectroscopy of dissolved crystals indicated that guinea pig hemoglobin crystallized as met-hemoglobin [27]. The active site and its surrounding amino acids are typical in comparison with other hemoglobins in the met state.

### Quaternary structure

Hemoglobins in the met-state usually crystallize in the R-state conformation and therefore a R-state conformation of the hemoglobin molecule was also expected for guinea pig met-hemoglobin. Unexpectedly the C-terminal four amino acids of the α-subunits had a very different conformation than normally found in the R-state. Superposition of guinea pig hemoglobin with human hemoglobin in T-, R- or R2-state revealed that guinea pig hemoglobin crystallized in the R2-state (Fig. 1). The R2-state of human hemoglobin features the same special C-terminal conformation of the α-subunit as observed in guinea pig hemoglobin [10]. Until now the R2-state has been only observed in hemoglobin crystals grown under low salt conditions. Hence it has been suggested that the R2-state may be the physiologically relevant liganded end state structure and that the R-state is an intermediate trapped between the R2 and T structures by the high-salt crystallization conditions [10], [11], [12]. Why does guinea pig hemoglobin crystallize in the R2-state under high-salt conditions?

### Stabilization of the R2-state

**Figure 2:**
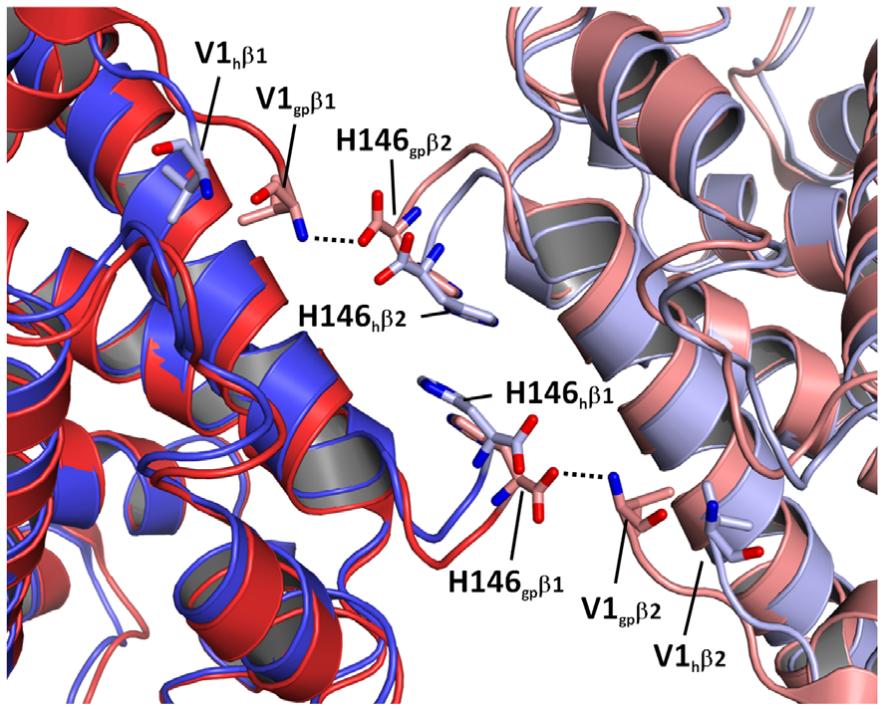
Stabilizing salt bridges of the β1/β2 interface in guinea pig hemoglobin. The β1/β2-interface of guinea pig hemoglobin in the R2-state is stabilized by two salt bridges between the N-terminal amino group of Val1 the β1-subunit and the C-terminal carboxyl group of the β2-subunit and vice versa. Both salt bridges are not present in the R2-state of human hemoglobin (1BBB)[10]. Guinea pig hemoglobin (β1 = red, β2 = light red) and human hemoglobin (β1 = blue, β2 = light blue) in the R2-state (PDB-code: 1BBB) were superimposed according to their C_α_-atoms. Carbon atoms of the N- and C-terminal amino acids of guinea pig hemoglobin are colored light red, while carbon atoms are colored blue in human hemoglobin. Oxygen and nitrogen atoms are colored red and blue respectively. Salt bridges are denoted by dotted lines.

One important factor that may contribute to crystallization in the R2-state is that the R2-state in guinea pig hemoglobin seems to be better stabilized than the R2-state in human hemoglobin. A comparison with R2-states of other species is not possible, since the R2-state has only been reported in human hemoglobin until now. Analysis of the salt bridges present in the tetramer reveals that guinea pig hemoglobin is stabilized by a total of 44 salt bridges in the R2-state, while human hemoglobin in comparison is only stabilized by 41 salt bridges in the R2-state. Out of these salt bridges both hemoglobins have 32 in common, while the remainder is unique to either one of them. The majority of salt bridges is formed between residues of the same subunit (intra-subunit salt bridges) and serves to stabilize a certain subunit. Only a few salt bridges stabilize interactions between adjacent subunits (intersubunit salt bridges) and thus are important for stabilization of the quaternary structure. Both, guinea pig and human hemoglobin, share one conserved salt bridge at the α1/β1-, α2/β2-, α1/β2- and α2/β1-interface in the R2-state. In contrast the β1/β2-interface of human hemoglobin in the R2-state is devoid of intersubunit salt bridges, while in guinea pig hemoglobin two intersubunit salt bridges are present (Fig. 2). They connect the N-terminus of the β1-subunit with the C-terminus of the β2-subunit and *vice versa*. Specifically they are formed between the N-terminal amino group (Val1) of one β-subunit and the C-terminal carboxyl group (His146) of the adjacent β-subunit. The distance between both charged groups is 2.6 Å. It was not possible to pinpoint a specific amino acid or set of amino acids responsible for the slightly different conformations of the C- and N-termini of the β-subunits in human and guinea pig hemoglobin. Nevertheless, we propose that the two salt bridges at the β1/β2-interface are an important factor, which increases the stability of the R2-state of guinea pig hemoglobin in comparison to human hemoglobin.

### Destabilization of the T-state

Besides stabilization of the R2-state an additional factor that might contribute to the oxygen binding behavior of guinea pig hemoglobin might be a destabilization of the T-state. Baldwin and Chothia described in their analysis of the hemoglobin tetramer flexible joint and switch regions [9]. The switch region involves residues 38–44 of the αl-subunit (C helix and CD corner) together with residues 97–102 of the β2-subunit (FG corner and G helix). In the course of the conformational change His97 of the β2-subunit slides in its position along helix C of the α1-subunit. Specifically, in the T-state His97 of the β2-subunit is positioned between Thr4l and Pro44 of the αl-subunit. Upon transition to the R-state, His97 of the β2-subunit moves one turn along the C helix of the α1-subunit to a position between residues Thr38 and Thr41 [9]. In the R2-state His97 of the β2-subunit slides even further in the same direction and is positioned opposite to Thr38 of the α1-subunit [10]. In human hemoglobin the switch region of the α1-subunit is stabilized by a salt bridge between Glu30 and His50, which connects helix C and helix E.

**Figure 3:**
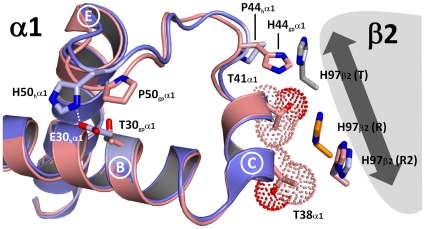
The “Switch” region of the α1/β2 interface in guinea pig hemoglobin. The switch region at the α1/β2 interface in guinea pig hemoglobin shows two important differences in comparison with human hemoglobin in the R2-state. Firstly, the stabilizing salt bridge, which connects Glu30 and His50 in human hemoglobin, is missing due to an amino acid exchange in guinea pig hemoglobin. Secondly, a steric hindrance between His97 of the β2-subunit and His44 of the α1-subunit might render the T-state of guinea pig hemoglobin less stable than the T-state in human hemoglobin, which has a Pro44 in the α1-subunit. Due to this steric hindrance a relaxed state conformation (R- or R2-state) of guinea pig hemoglobin could be favored, thereby increasing its oxygen affinity. The α1- and β2-subunit of guinea pig hemoglobin (α1 = light red) and human hemoglobin (α1 = light blue) in the R2-state (PDB-code: 1BBB, [10]) were superimposed according to their C_α_-atoms. Carbon atoms of amino acids in guinea pig hemoglobin are colored light red, while the carbon atoms are colored blue in human hemoglobin. The position of the β2-subunit is denoted by a light grey area, while the sliding movement of His97 in the course of the conformational transition is illustrated by an arrow (dark grey). The position of His97 in the R2-state of guinea pig hemoglobin is colored in light red. Furthermore the position of His97 in human hemoglobin is shown in the T-state (grey, PDB-code: 1A3N, [36]), R-state (orange, PDB-code: 1HHO, [37]) and R2-state (light blue, PDB-code: 1BBB, [10]).

In the switch region of guinea pig hemoglobin two important differences exist in comparison to human hemoglobin (Fig. 3). Firstly, the salt bridge stabilizing the switch region in human hemoglobin is not present due to two amino acid exchanges (Glu30Thr30 and His50Pro50) in the α1-subunit of guinea pig hemoglobin (Fig. 3). The absence of this salt bridge most probably renders the switch region more flexible in guinea pig hemoglobin. Secondly, in the T-state His97 of the β2-subunit in human hemoglobin is positioned opposite to Pro44 of the α1-subunit. In guinea pig hemoglobin a bulky histidine instead of proline is found at position 44 of the α1-subunit, which inevitably will result in a steric hindrance between His44 and His97 of the β2-subunit (Fig. 3). This steric hindrance renders the T-state of guinea pig hemoglobin less stable and therefore will result in a higher oxygen affinity since the equilibrium between T-, R- and R2-state will be shifted towards the relaxed states (either R- or R2-state), which have a higher oxygen affinity. Human hemoglobin mutants confirm this hypothesis, since the mutants Milledgeville (Pro44Leu) and Kawachi (Pro44Arg) have a strongly increased oxygen affinity [33], [34]. This may explain why Pro44 of the α-subunit is highly conserved in vertebrate hemoglobin. Exceptions are hemoglobins of rat (*Rattus norvegicus*) and several fish species which have exchanged Pro44 of the α-subunit to a serine. However, no increased oxygen affinity has been reported for the exchange of Pro44Ser most probably due to the fact that serine is much smaller than leucine, arginine or histidine and therefore no steric hinderance exists with His97 of the β2-subunit.

In conclusion we propose that the increased oxygen affinity of guinea pig hemoglobin can be explained by two factors, namely a decreased stability of the T-state and an increased stability of the R2-state. Both factors together serve to shift the equilibrium between the conformational states towards the high affinity relaxed states resulting in an increased oxygen affinity of guinea pig hemoglobin. It is remarkable that guinea pig hemoglobin crystallizes in the R2-state under oxy high salt conditions, which in all other vertebrate hemoglobins provokes formation of crystals in the R-state. This may indicate that the R2-state in guinea pigs has an increased stability and thus is the physiological relaxed state, while in human hemoglobin the R-state or a mixture of R- and R2-state seems to be present as the relaxed state [13]. Surely the increased oxygen affinity of hemoglobin is not the only adaptation to high altitude in guinea pigs, but increased blood oxygen affinity has been proven to be advantageous for animals living at high altitudes, because it increases the oxygen saturation of the arterial blood [1], [2], [3], [19], [35].
